# Rural Villagers and Urban Residents Exposure to Poultry in China

**DOI:** 10.1371/journal.pone.0095430

**Published:** 2014-04-25

**Authors:** Zhibin Peng, Peng Wu, Li Ge, Richard Fielding, Xiaowen Cheng, Weike Su, Min Ye, Ying Shi, Qiaohong Liao, Hang Zhou, Lei Zhou, Leilei Li, Jiabing Wu, Shunxiang Zhang, Zhangda Yu, Xiaomin Wu, Hanwu Ma, Jianhua Lu, Benjamin J. Cowling, Hongjie Yu

**Affiliations:** 1 Division of Infectious Disease, Key Laboratory of Surveillance and Early-warning on Infectious Disease, Chinese Center for Disease Control and Prevention, Beijing, China; 2 University of Hong Kong, Hong Kong, Special Administrative Region, China; 3 Xiangyang Center Hospital, Xiangyang, China; 4 Shenzhen Center for Disease Control and Prevention, Shenzhen, China; 5 Xiuning Center for Disease Control and Prevention, Xiuning, China; 6 Zhoushan Entry-exit Inspection and Quarantine Bureau, Zhoushan, China; 7 Public Health Emergency Center, Chinese Center for Disease Control and Prevention, Beijing, China; 8 Anhui Provincial Centers for Disease Control and Prevention, Hefei, China; 9 Huanshan Center for Disease Control and Prevention, Huangshan, China; The Australian National University, Australia

## Abstract

Patterns of poultry exposure in rural and urban areas in China have not been systematically evaluated and compared. The objective of our study is to investigate patterns in human exposure to poultry in rural and urban China. We conducted a two-stage household-based clustered survey on population exposure to live/sick/dead poultry in Xiuning and Shenzhen. Half of the rural households (51%) in Xiuning raised poultry, mostly (78%) free-range. Around half of those households (40%) allowed poultry to stay in their living areas. One quarter of villagers reported having contact with sick or dead poultry. In Shenzhen, 37% urban residents visited live poultry markets. Among these, 40% purchased live poultry and 16% touched the poultry or cages during purchase. Our findings indicated that human exposure to poultry was different in rural and urban areas in China. This discrepancy could contribute to the observed differences in epidemiologic characteristics between urban and rural cases of influenza A(H7N9) and A(H5N1) virus infection.

## Introduction

The highly pathogenic avian influenza (HPAI) virus, A (H5N1) [1], caused 630 confirmed human cases and 375 deaths in 15 countries as of June 4, 2013 [2]. Despite widespread human exposure to H5N1-infected poultry [3], [4], confirmed human H5N1 infections remain rare [5]. Avian-to-human transmission of H5N1 virus is believed to have occurred in most human cases [2], with rare instances of limited, non-sustained human-to-human H5N1 virus transmission [6], [7], [8]. Environment-to-human transmission remains a possibility for some human H5N1 cases without an identified exposure source [5], [9]. Case-control studies and case investigations conducted during the 1997 outbreak in Hong Kong Special Administrative Region (SAR) of China [10], Thailand [11], Vietnam [12], Indonesia [5], [7], [9], Egypt [13] and Mainland China [14] during 2004–2009 revealed that visiting a live poultry market and direct contact with sick or dead poultry one week before illness onset were risk factors for H5N1 virus infection.

Of the 37 confirmed human H5N1 cases reported in China, 36 were identified through surveillance systems [15] from October 2005 [16] through August 2009 [2]. One additional H5N1 case was detected in 2003 [17]. These cases occurred sporadically, and included 19 urban cases and 24 rural cases were distributed among 21 counties and 16 districts of 17 provinces, with no obvious geographical clustering. In China, two patterns of exposure to birds were identified among the human cases: in rural environments, villagers raise backyard poultry for food production and income and exposure is primarily associated with the backyard poultry, whereas in urban areas exposure primarily occurs in live poultry markets where poultry is purchased alive or freshly slaughtered [14], [18].

On March 31^st^ 2013, the National Health and Family Planning Commission (NHFPC) of China reported severe acute respiratory disease caused by a novel avian influenza A (H7N9) virus [19]. By 31 May 2013, 131 cases were reported in China, which included 93 cases in urban areas and 37 cases in rural areas [20]. According to available evidence, infections mainly occurred through close contact with infected poultry, or proximity to environments contaminated by the virus such as live poultry markets [21], [22], [23], [24], [25].

As part of a comprehensive program on identifying risk factors associated with human avian influenza virus infection in China, we evaluated exposure to backyard poultry in rural villagers, and poultry exposure in live poultry markets in an urban area, to investigate and compare the patterns of exposure to poultry in rural and urban populations.

## Materials and Methods

This study was carried out in one rural area – Xiuning County, and one urban area – Shenzhen, in China in 2007. Xiuning County is a rural county located in Anhui Province in central China. Shenzhen is a city in Guangdong Province, in the southeast of China, bordering Hong Kong, SAR. Confirmed human H5N1 cases were reported in Xiuning and Shenzhen on November 2005 [26] and June 2006 [27], respectively. These two areas were chosen because of the recent reports of human H5N1 cases in each area, and based on consideration of feasibility and accessibility.

### Study Design

We conducted a two-stage household-based clustered survey to assess exposures potentially associated with human avian influenza in each area using probability proportional to size (PPS) sampling techniques. The sampling method is described in more detail in Text S1, and the sampling frame in Shenzhen is shown in Table S1 in Text S1. Trained investigators from the Chinese Center for Disease Control and Prevention (China CDC, Beijing) and local CDCs described the purpose of the study to eligible subjects or their proxies and obtained verbal informed consent. In each household, every family member who met the inclusion criteria was interviewed. The inclusion criteria were a) ability to communicate, b) ≥5 years of age, and c) resident in the study area for ≥3 months.

The sample size calculated for the survey in Shenzhen was 1,750 participants to allow for the type I error to be 0.05, based upon an estimated prevalence of live chicken purchase behavior among Hong Kong residents to be 20% [28]. We estimated that 2700 subjects were needed in Xiuning to allow for 50% of households raising backyard poultry. In both settings, the sample size calculation took into account different sampling methods and assumed a response rate of 80%.

A live poultry market was defined as a place where small animals and poultry are purchased alive or slaughtered just before purchase [29]. Direct contact with poultry was defined as handling live poultry with bare hands. Poultry was defined as domesticated birds kept by humans for the purpose of collecting of eggs, or killing for meat or feathers, including chickens, domestic ducks, domestic geese, quails and turkeys, etc.

A standardized questionnaire was used to collect information on demographics, including sex, age, education, occupation, frequency of visits to live poultry markets for urban residents (Shenzhen), and exposure to sick or dead poultry for rural villagers (Xiuning). In rural households, one female family member representing the household was asked about backyard poultry raising (presence of any backyard poultry; number of fowl; if allowed to enter the house; poultry vaccination), and all household members were asked about touching sick/dead poultry directly (yes/no/unclear), subsequent hand-washing practices (yes/no/unclear) and disposal of sick/dead poultry. Urban residents were asked about their live poultry purchasing behaviors, their frequency of live poultry market visit and their frequency of touching a bird/bird cage while purchasing, and by whom and where the bird was slaughtered (exposure questions). Subjects who answered touching “sometimes” or “frequently” were asked to specify the frequency of subsequent hand contact with the mouth, nose or eye to determine the potential risk of self-inoculation.

The surveys were carried out between July 21 and September 8, 2007 in Xiuning, and from August 9 to August 30 in Shenzhen. All questions were answered by individual participants in a household, or a household representative. Child participants (5–17 years of age) were allowed to answer questions with the help from investigators or parents to rephrase/explain the question while encountering difficulty in understanding it. If participants in the selected households were not available at the first visit, up to two further visits were attempted within 48 hours to recruit eligible persons. Anyone who refused to participate or who was not available after three attempts was excluded.

Neither interviewees nor interviewers were informed about the purpose of the research. Field supervisors from China CDC led the study, trained the interviewers, and re-checked all questionnaires. Five percent respondents were randomly selected to be re-interviewed within a week after the first interview. Consistency between two interviews was greater than 90%.

### Statistical Analyses

Data extracted from questionnaires were double-entered and verified using EpiData software (Odense, Denmark; accessed at: http://www.epidata.dk/links.htm). All analyses were conducted using SPSS (version 13.0, SPSS Inc., Chicago, Illinois, United States) and Stata/IC version 10.1 (Stata Corporation, College Station, Texas United States). The median and the range were provided for continuous variables, and compared between the urban and rural groups using the Wilcoxon rank sum test. For categorical variables, urban and rural groups were compared using the chi-squared test.

Average annual numbers of live poultry market visits and live poultry purchasing rates were calculated by using a conservatively estimated number of visits or purchases per response category [3], [30]. For respondents reporting ≤1 live poultry market visit or live poultry purchase in a year, we counted their yearly visit/purchase as 1, 4 for those reporting frequency of 3–5 visits/year, 8 for 6–11 visits/year; 24 for 1–3 visits/month, 52 for 1–2 visits/week, 208 for 3–5 visits/week, and 365 for ≥1 visit/day. Participants who answered “frequent” or “sometimes” in questions regarding poultry contact were treated as having had direct contact with poultry in the analyses.

All statistical tests were two-sided with a type I error being 0.05.

### Study Approval

The study was approved by the Institutional Review Board of China CDC and was carried out in compliance with the Helsinki Declaration. The requirement for signed informed consent was waived because no sensitive individual information or clinical specimens were collected from participants. Verbal informed consent was obtained from the participants ≥14 years of age or above or parents/guardians for children (5–13 years of age).

## Results

### Demographic Characteristics

A total of 4,966/5,033 (99%) eligible people participated in the study and completed the questionnaire (Table 1). Fifteen (<1%) participants in Shenzhen and one (<1%) in Xiuning were excluded from analysis due to missing data on exposure, leaving a total of 4,950 in the analyses. Among the 4,950 participants, 2,058 were from 994 Shenzhen households and 2,892 villagers were from 1,053 Xiuning households. Respondents in Shenzhen were younger than those in Xiuning, while more elderly people were observed in Xiuning (p<0.001). The urban group had higher levels of education than the rural group (p<0.001). Household size was significantly different between urban and rural groups (p<0.001).

**Table 1:** Demographic characteristics of residents in Shenzhen and Xiuning in China in 2007.

	Shenzhen (%)	Xiuning (%)	Total (%)
	(n = 2,058)	(n = 2,892)	(N = 4,950)
Male ‡	1,003 (49)	1,396 (48)	2,399 (48)
Median age* (years, range)	30 (5–83)	44 (5–94)	37 (5–94)
Age group‡			
5–18	304 (15)	430 (15)	734 (15)
19–39	1,236 (60)	754 (26)	1,990 (40)
40–59	425 (21)	1150 (40)	1575 (32)
≥60	93 (4)	558 (19)	651 (13)
Highest level of education‡			
None or kindergarten	122 (6)	626 (22)	748 (15)
Primary school	417 (20)	1,228 (42)	1,645 (33)
Junior high school	759 (37)	839 (29)	1,598 (32)
High school	466 (23)	182 (6)	648 (13)
College or higher	294 (14)	17 (1)	311 (7)
Occupation‡			
Employed	1,268 (62)	2,282 (79)	3,550 (72)
Unemployed	144 (7)	108 (4)	252 (5)
Students	296 (14)	441 (15)	737 (15)
Homemakers	244 (12)	39 (1)	283 (6)
Retired	106 (5)	22 (1)	128 (2)
Household size	n = 994	n = 1,053	N = 2,047
Median (range)*	2 (1–11)	3 (1–9)	2 (1–11)
1	353 (36)	155 (15)	508 (25)
2	379 (38)	340 (32)	719 (35)
3	158 (16)	290 (28)	448 (22)
4	71 (7)	180 (17)	251 (12)
≥5	33 (3)	88 (8)	121 (6)
Household with children <5 years‡	168 (17)	136 (13)	304 (15)
Household with children <15 years‡	296 (30)	368 (35)	664 (32)

*Median age and household size between urban and rural groups were compared by the Wilcoxon rank sum test, Z = 12.137, P<0.001 and Z = −13.664, P<0.001.

‡ Frequencies for male, age group, highest level of education, occupation status, household with children <5 years and household with children <15 years between urban and rural group were compared by the Chi-squared test, with p = 0.747, p<0.001, p<0.001, p<0.001, p = 0.016 and p = 0.013, respectively.

### Rural Household Exposure to Backyard Poultry

Overall, 532/1,043 (51%) rural households reported raising backyard poultry, with 508/532 (95%) having less than 20 animals. Most 411/530 (78%) raised free-range poultry with only 119/530 (22%) keeping their poultry cooped (Table 2). Animals were often (40%) or seldom (13%) allowed to go into the household living areas. The remainder (47%) kept their poultry outdoors. In China, a national compulsory H5N1 poultry vaccination program was implemented in December 2005 [31]. In our sample only 38% (95% confidence interval (CI) 34–42%, 196/515) of the villagers had all of their animals vaccinated, while 51% (95% CI 47–55%, 262/515) never vaccinated their poultry. 24% (95% CI 21–27%, 127/532) households raising poultry reported having sick or dead poultry before. Of these, only 2% (2/127) reported this to the local authorities. Of the 127 households that had sick or dead poultry, 57% (49–66%) discarded the dead animals, 38% (30–46%) buried them, and 3% (0–6%) consumed them.

**Table 2:** Backyard poultry* raising among 1,053 households in Xiuning County, China, 2007.

Characteristics	No. (%)†
Households with backyard poultry	532 (51)
Number of poultry raising Median (range)	7 (1–800)
1–5	208 (39)
6–10	184 (34)
11–20	116 (22)
21–800	24 (5)
Type of raising	
Free range raising	411/530 (78)
Raising in cages	119/530 (22)
Live poultry ever enters home	
Often	214/529 (40)
Seldom	69/529 (13)
Never	246/529 (47)
Poultry H5 vaccination coverage	
All	196/515 (38)
More than 50%	24/515 (5)
Less than 50%	33/515 (6)
None	262/515 (51)
Experienced sick or dead poultry	127/532 (24)
Reporting sick or dead poultry to local authority	2/127 (2)
Disposal of sick/dead poultry	
Discarded	73/127 (57)
Buried or burned	48/127 (38)
Consumed	4/127 (3)
Unknown	2/127 (2)

*Including chicken, ducks, geese and other domestic birds.

† Denominators smaller than the complete sample were indicated.

Of the total 2,892 villagers, 5.0% (95% CI 4.2–5.8%, 144/2,892) respondents reported directly contact with sick/dead poultry (gender adjusted). There was no statistical significance between male and female in contacting sick/dead poultry during the past year (*P* = 0.664). 2.1% (95% CI 0.8–3.5%, 10/468) of 5–18 years respondents reported direct contact with sick/dead poultry, 5.4% (95% CI 4.3–6.4%, 100/1,866) and 6.1% (95% CI 4.1–8.1%, 34/558) in 19–59 years and ≥60 years, respectively (Table 3). The prevalence of contact reported in the three age groups was statistically different (*P* = 0.007). Respondents aged 19–59 years more often contacted sick/dead poultry than those aged 5–18 years (*P* = 0.003), and ≥60 years contacted sick/dead poultry more often than 5–18 years (*P* = 0.002). However the contact difference between 19–59 years and ≥60 years was not statistically significant (*P* = 0.506).

**Table 3:** Age-specific poultry exposure among 2,892 villagers in Xiuning, China, 2007.

	Male (n = 1396)			Female (n = 1496)		
	No. (%)			No. (%)		
	5–18	19–59	≥60	5–18	19–59	≥60
	(n = 245)	(n = 895)	(n = 256)	(n = 223)	(n = 971)	(n = 302)
Households with backyard poultry	123 (50)	465 (52)	141 (55)	106 (48)	509 (52)	165 (55)
Live poultry ever enters home						
Often	55 (22)	174 (19)	67 (26)	42 (19)	199 (20)	66 (22)
Seldom	14 (6)	72 (8)	13 (5)	10 (4)	66 (7)	22 (7)
Never	54 (22)	219 (24)	60 (23)	54 (24)	243 (25)	75 (25)
Experienced sick or dead poultry	26 (11)	110 (12)	34 (13)	24 (11)	134 (14)	37 (12)
Directly contact with sick/dead poultry	6 (2)	45 (5)	16 (6)	4 (2)	55 (6)	18 (6)

Frequencies of households reporting raising backyard poultry, allowing live poultry to enter the home, having experienced sick or dead poultry, and having direct contact with sick/dead poultry were compared between three age groups by Chi-squared test, p = 0.169, p = 0.220, p = 0.217 and p = 0.007.

We also estimated exposure to poultry among the entire population in Xiuning County where 240, 000 population lived in 75,000 households [32]. Adjusting for household size, the prevalence of poultry raising in Xiuning population was 52% (95% CI 48–56%) (Table S2 in Text S1), representing 38,000 (95% CI 35,000–42,000) households in 2007, and 13% (Table S3 in Text S1) of the villagers, equivalent to 9,586 households, would have had sick/dead poultry identified in their backyard in Xiuning County. 144/2,892 respondents had contacted sick/dead poultry in the past year, which implied that approximately 12,000 persons might have contacted sick/dead poultry during the period in Xiuning County.

### Exposures in Urban Live Poultry Markets

Overall, 1,284/2,058 urban participants, (62%; 95% CI 60–64%) never visited live poultry markets (Table 4). Of the remainder (774/2,058; 38%; 36–40%) who had ever visited live poultry markets in the past year, live poultry market visiting frequency was statistically different among three age groups (*P*<0.001). Those aged 19–59 years and ≥60 years more often visited live poultry markets than aged 5–18 years (*P*<0.001, and *P*<0.001, respectively), but the difference between 19–59 years and ≥60 years was not statistically significant (*P* = 0.102). Twice as many women as men visited live poultry markets during the past year (*P*<0.001). 40% (393/994) of the sampled households purchased live poultry, and 17% households purchased 1–3 live poultry/month.

**Table 4:** Frequency of live poultry market visits among 2,058 residents in Shenzhen, China, 2007.

	Male (n = 1003)			Female (n = 1055)			Total No. of visits in subjects selected in the survey
	No. (%)			No. (%)			
	5–18	19–59	≥60	5–18	19–59	≥60	
	(n = 198)	(n = 767)	(n = 38)	(n = 151)	(n = 849)	(n = 55)	
Never visited	151 (74)	522 (68)	23 (60)	124 (83)	436 (52)	28 (51)	–
Visited*							
≤1/year	7 (4)	14 (2)	1 (3)	3 (2)	26 (3)	0 (0)	51
3–5/year	9 (5)	41 (5)	0 (0)	3 (2)	59 (7)	3 (5)	460
6–11/year	3 (2)	12 (2)	1 (3)	1 (1)	9 (1)	1 (2)	216
1–3/month	16 (8)	64 (8)	2 (5)	8 (5)	100 (12)	5 (9)	4,680
1–2/week	3 (2)	39 (5)	3 (8)	5 (3)	62 (7)	2 (4)	5,928
3–5/week	2 (1)	15 (2)	1 (3)	2 (1)	26 (3)	3 (5)	10,192
1/day	7 (4)	60 (8)	7 (18)	5 (3)	131 (15)	13 (24)	81,395
Subtotal visited	47 (26)	245 (32)	15 (40)	27 (17)	413 (48)	27 (49)	102,922
Average annual visits among residents							50
Adjust average annual visits among residents§							47.2

*Standardized number of visiting per unit time: 1 was attributed to reporting ≤1 live poultry market visit per year; 4 to reporting 3–5 times/year; 8 to reporting 6–11 times/year; 24 to reporting 1–3 times/month; 52 to reporting 1–2 times/week; 208 to reporting 3–5 times/week; and 365 to those reporting ≥1/day annually.

§ The average annual household visits was weighted by age and sex based on the National Census in 2000.

We estimated that there were approximately 470 (350–580) million visits to live poultry markets among 9.9 million populations in Shenzhen during 2007 through extrapolation using the population size and the number of households in the city during the same period [33]. In total, around 41 million live poultry purchases occurred in the past year, and there were approximately 640,000 person-exposures in Shenzhen in 2007 based on the estimate from the study that 6.4% (132/2,058) of participants touched poultry (gender-adjusted 6.42%) while purchasing.

Self-inoculation after touching live poultry or cages was reported by 1.1% (23/2,058) or 0.4% (9/2,058) of participants, respectively, with gender-adjusted proportion to be 1.05% (males: 1.1%; females: 1.0%) or 0.5% (males: 0.4%; females: 0.6%). Based on this we extrapolated approximately 100,000 and 45,000 potential self-inoculations/year in Shenzhen in 2007.

## Discussion

China CDC has investigated factors influencing the risk of exposure to avian influenza, established patterns of transmission, and provided preventive recommendations [8], [14], [15], [18]. By comparing rural Xiuning County, where raising backyard poultry was common, with urban Shenzhen, where residents regularly visited live poultry markets, we assessed the nature and extent of exposure to poultry in the two settings.

In our study, 51% of rural households raised poultry, half of them reporting episodes of sick or dead poultry, but only 2% notified the local authorities. In comparison, Thorson *et al* found 21% of a rural population sample in Vietnam raised or kept poultry in their households; of those, 27% reported having sick or dead poultry [33]. In another study conducted in Vietnam, 22% of 1,150 backyard poultry keepers reported sickness or deaths of their poultry, only 9% informing the authorities [3]. In the urban setting of our survey, 63% of respondents, undifferentiated by gender, reported that they had never visited live poultry markets. A much lower percentage was reported in Hong Kong [28] where 20% of females and 27% of males reported that they had never purchased live poultry in live poultry markets. These different rates may reflect lower perceived risks of HPAI in Hong Kong, which has remained free of HPAI cases since 2002, compared to China where there have been more recent and regular HPAI cases [2], [14], [30]. A previous study also showed that higher perceived risk to HPAI might be associated with lower live poultry purchases [30].

For urban households, if each human–poultry contact when buying poultry is counted as a potential exposure, then 2.7 million exposures per year, or 0.27 potential exposures/year/person occurred. A Hong Kong telephone survey in 2006 similarly reported 0.23 market-related exposures/year/person [28]. For rural households, assuming conservatively that all persons within these households have at least weekly physical contact with their birds, bird eggs, or feces, and knowing that household size in the surveyed districts averages 3.2 persons, 6.5 million exposures/year would occur from backyard poultry in the surveyed districts, equivalent to an average of 27 exposures/person/year. If daily backyard exposure occurred, then there would be around 45 million exposures, or 187 exposures/person/year. Comparable results were reported from Vietnam (27.6 and 194 exposures/person/year, respectively) [3].

Different exposure patterns might provide evidence for observed characteristics of human avian influenza virus infections, including age, sex, and geographic distributions of patients. In comparison with H5N1 cases, more H7N9 cases were reported in elderly men in urban areas, which might be due to higher exposure to live poultry markets in these population, greater prevalence of underlying conditions associated with severe disease if infected, or other reasons [34].

There are a few limitations of the study. First, we did not estimate and could not rule out potential exposure to avian influenza via other sources, such as poultry feces [3]. Second, the subject was asked in the survey to report poultry contact and related behaviors in the past year, and this could have been affected by recall bias or reporting bias. Third, there may have changes over time in population’s purchase behavior or routine contact patterns with backyard poultry in rural area which could be addressed in further longitudinal studies. Finally, our sample may not fully represent the behaviors of urban and rural populations in other areas of China, and future studies could explore geographical heterogeneity in exposure patterns.

## Conclusions

The findings from this study indicated that characteristics of exposure to poultry were very different between urban and rural populations in China. The varied contact patterns could explain patterns in cases infected with H5N1 and the novel H7N9 virus in China given that contact with poultry has been considered the most important risk factor leading to infection [33]. Public interventions to reduce exposure to live/sick/dead poultry might be warranted both among urban and rural residents particularly in high-risk groups in order to decrease the risk of infection with avian influenza. In the short term, the influenza H5N1 vaccination programme in China should be stepped up to reduce potential infection with and transmission of the virus. In the longer term, establishing and improving central slaughtering facilities in China particularly in urban areas can help reduce population exposure to avian influenza virus by substantially decreasing contact with live poultry.

## Supporting Information

Text S1 Description of supplementary information. Table S1: Illustration of information collected for selection of primary sampling unit in the sampling for the survey in Shenzhen. Table S2: Standardization of backyard poultry exposure estimated from the survey conducted in Xiuning. Table S3: Standardization of sick/dead poultry exposure estimated from the survey conducted in Xiuning.
(DOC)
