# Supplementary material for: Rural Villagers and Urban Residents Exposure to Poultry in China
Source: PLoS One. 2014 Apr 25;9(4):e95430. doi: 10.1371/journal.pone.0095430 (PMC4000224; doi:10.1371/journal.pone.0095430)
Supplement: Text S1 — Description of supplementary information. Table S1: Illustration of information collected for selection of primary sampling unit in the sampling for the survey in Shenzhen. Table S2: Standardization of backyard poultry exposure estimated from the survey conducted in Xiuning. Table S3: Standardization of sick/dead poultry exposure estimated from the survey conducted in Xiuning. (DOC) [file pone.0095430.s001.doc]

**Rural villagers and urban residents exposure to poultry in China**

**Supplementary Information**

Zhibin Peng^1^, Peng Wu^2^, Li Ge^3^, Richard Fielding^2^, Xiaowen Cheng^4^, Weike Su^5^, Min Ye^6^, Ying Shi^1^, Qiaohong Liao^1^, Hang Zhou^1^, Lei Zhou^7^, Leilei Li^7^ Jiabing Wu^8^, Shunxiang Zhang^4^, Zhangda Yu^9^, Xiaomin Wu^4^, Hanwu Ma^4^, Jianhua Lu^4^, Benjamin J. Cowling^2^, Hongjie Yu^1^

^1^ Division of Infectious Disease, Key Laboratory of Surveillance and Early-warning on Infectious Disease, Chinese Center for Disease Control and Prevention, Beijing, China

^2^ University of Hong Kong, Hong Kong, Special Administrative Region, China

^3^ Xiangyang Center Hospital, Xiangyang, China

^4^ Shenzhen Center for Disease Control and Prevention, Shenzhen, China

^5^ Xiuning Center for Disease Control and Prevention, Xiuning, China

^6^ Zhoushan Entry-exit Inspection and Quarantine Bureau, Zhoushan, China

^7^ Public Health Emergency Center, Chinese Center for Disease Control and Prevention, Beijing, China

^8^Anhui Provincial Centers for Disease Control and Prevention, Hefei, China

^9^Huanshan Center for Disease Control and Prevention, Huangshan, China

**1. Sampling method of probability-proportional to size (PPS)**

We randomly selected 30 communities in Shenzhen (urban area) or villages in Xiuning (rural area) with the probability-proportional to size method in this survey. About 20 households per community and 36 households per village were needed in order to meet the sample size.

Sampling process:

- Urban area-Shenzhen:

In order to meet the sample size requirement for Shenzhen (1,750 subjects), we needed around 600 households recruited in the city with the average household size estimated for the community to be 2.97. The sampling process includes two steps as follows:

Step 1: Sampling in community

1. We obtained geographic locations and population size for each community in Shenzhen. List the community name within 6 districts based on letter order and east, west, north and south order. Get total population and accumulative population for each community under local people’s help.
2. Each community was treated as a primary sampling unit (PSU). We constructed the table for selection of PSU by including the name of the community, the district to which the community belongs, population size in each community, cumulative population size, and random numbers. (Supplementary Table 1)

Table S1. Illustration of information collected for selection of primary sampling unit in the sampling for the survey in Shenzhen.

| PSU No. | Name of District | street | Name of Community | Population | Cumulative population | Random Number* |
| --- | --- | --- | --- | --- | --- | --- |
| 1 | Bao An | Xin An | Bao Min | 10000 | 10000 | … |
| 2 |  |  |  | 15000 | 25000 | … |
| 3 |  |  |  | 5000 | 30000 | R |
|  |  |  |  | 15000 | 45000 | … |
| … |  |  |  | … | … | … |
| 25 |  |  |  | … | … | R+K |
|  |  |  |  |  |  |  |

*The row where the random number locates in the table indicates that the PSU (community) in the same row was selected

3) We derived the sampling interval, K, equals to the number of total population in ShenZhen divided by the number of selected PSUs.

4) A random number from 1 to K (equals to 409,518) was generated as the random start (R). If the random number generated is smaller than or equal to K, then we use it as R, while we use the remainder of the random number divided by K as R instead if the random number is larger than K. Thirty numbers were generated by using the following formula: R;R+K; R+2K; R+3K;..., R+29K.

5) Selection of PSU

We compared the 30 numbers generated in 4) with the cumulative population sizes in Table 1, and selected the community with the cumulative population size including the random number.

Step 2: Selection of households

Twenty households in each selected community were randomly selected with a computer-generated random number from 1 to the total number of households in the community. An alternative household nearest to the originally selected household would be recruited if the original household refused to participate in the survey or was not available.

- Rural area-Xiuning:

We used a similar PPS method as that applied in Shenzhen survey to select households in Xiuning. The differences include that villages were used as PSUs in the rural area, 30 villages were finally selected, and 36 households were recruited in each selected village.

**2. Statistical methods for estimation of poultry exposure in Xiuning**

We used the age and sex distribution of the Chinese population reported by the National Census conducted in 2000 which is the only available reference dataset providing detailed information on household size and sex ratio at county level for extrapolation.

Table S2. Standardization of backyard poultry exposure estimated from the survey conducted in Xiuning.

|  | The reference population | | | Our sample | | |
| --- | --- | --- | --- | --- | --- | --- |
| Household size | No. of household | Percentage  (%) |  | No. of household | The percentage of household  with poultry (%) | Adjusted  Percentage (%) |
| 1 | 7,772 | 10.30 |  | 155 | 38.71 | 3.99 |
| 2 | 15,795 | 20.93 |  | 340 | 52.06 | 10.90 |
| 3 | 22,885 | 30.32 |  | 290 | 51.03 | 15.48 |
| 4 | 17,809 | 23.59 |  | 180 | 56.11 | 13.24 |
| 5+ | 11,221 | 14.87 |  | 88 | 53.41 | 7.94 |
| Total | 75,482 | 100 |  | 1,053 |  | 51.53 |

Table S3. Standardization of sick/dead poultry exposure estimated from the survey conducted in Xiuning

|  | The reference population | | | Our sample | | |
| --- | --- | --- | --- | --- | --- | --- |
| Household size | No. of household | Percentage  (%) |  | No. of household | Proportion of households with exposure to sick/dead poultry (%) | Adjusted  Percentage (%) |
| 1 | 7,772 | 10.30 |  | 155 | 7.74 | 0.80 |
| 2 | 15,795 | 20.93 |  | 340 | 11.76 | 2.46 |
| 3 | 22,885 | 30.32 |  | 290 | 13.10 | 3.97 |
| 4 | 17,809 | 23.59 |  | 180 | 16.11 | 3.80 |
| 5+ | 11,221 | 14.87 |  | 88 | 11.36 | 1.69 |
| Total | 75,482 | 100 |  | 1,053 |  | 12.72 |

The stratum-specific adjusted percentages were calculated by multiplying the percentage of households with poultry in the sample and corresponding percentage in the reference population divided by 100, e.g. 3.98= (38.71*10.30)/100. The total adjusted percentage was the sum of each stratum-specific adjusted percentages. All other proportions were estimated in the similar manner in this study.
